# Zucker Diabetic‐Sprague Dawley Rats Have Impaired Peri‐Implant Bone Formation, Matrix Composition, and Implant Fixation Strength

**DOI:** 10.1002/jbm4.10819

**Published:** 2023-10-11

**Authors:** Kyle D Anderson, Christian Beckmann, Saskia Heermant, Frank C Ko, Bryan Dulion, Imad Tarhoni, Jeffrey A Borgia, Amarjit S Virdi, Markus A Wimmer, D Rick Sumner, Ryan D Ross

**Affiliations:** ^1^ Department of Anatomy and Cell Biology Rush University Medical Center Chicago IL USA; ^2^ Department of Orthopedic Surgery Rush University Medical Center Chicago IL USA; ^3^ Department of Microbial Pathogens and Immunity Rush University Medical Center Chicago IL USA

**Keywords:** ANIMAL MODELS, BIOMECHANICS, BONE MATRIX, DIABETES, IMPLANTS

## Abstract

An increasing number of patients with type 2 diabetes (T2DM) will require total joint replacement (TJR) in the next decade. T2DM patients are at increased risk for TJR failure, but the mechanisms are not well understood. The current study used the Zucker Diabetic‐Sprague Dawley (ZDSD) rat model of T2DM with Sprague Dawley (SPD) controls to investigate the effects of intramedullary implant placement on osseointegration, peri‐implant bone structure and matrix composition, and fixation strength at 2 and 10 weeks post‐implant placement. Postoperative inflammation was assessed with circulating MCP‐1 and IL‐10 2 days post‐implant placement. In addition to comparing the two groups, stepwise linear regression modeling was performed to determine the relative contribution of glucose, cytokines, bone formation, bone structure, and bone matrix composition on osseointegration and implant fixation strength. ZDSD rats had decreased peri‐implant bone formation and reduced trabecular bone volume per total volume compared with SPD controls. The osseointegrated bone matrix of ZDSD rats had decreased mineral‐to‐matrix and increased crystallinity compared with SPD controls. Osseointegrated bone volume per total volume was not different between the groups, whereas implant fixation was significantly decreased in ZDSD at 2 weeks but not at 10 weeks. A combination of trabecular mineral apposition rate and postoperative MCP‐1 levels explained 55.6% of the variance in osseointegration, whereas cortical thickness, osseointegration mineral apposition rate, and matrix compositional parameters explained 69.2% of the variance in implant fixation strength. The results support the growing recognition that both peri‐implant structure and matrix composition affect implant fixation and suggest that postoperative inflammation may contribute to poor outcomes after TJR surgeries in T2DM patients. © 2023 The Authors. *JBMR Plus* published by Wiley Periodicals LLC on behalf of American Society for Bone and Mineral Research.

## Introduction

The prevalence of type 2 diabetes mellitus (T2DM) and total joint replacement (TJR) in the United States is expected to rise to nearly 40 million^(^ ^1^ ^)^ and 1.5 million by 2030,^(^ ^2^ ^)^ respectively. The prevalence of TJR patients with T2DM is, thus, expected to increase. On average, 2% to 18% of patients undergoing TJR present with known T2DM, and 11% to 36% of TJR patients are diagnosed with previously unidentified T2DM during preoperative screening.^(^ ^3^, ^4^, ^5^ ^)^ Importantly, T2DM increases the risk for adverse outcomes,^(^ ^6^ ^)^ including late‐stage loss of implant fixation.^(^ ^7^, ^8^, ^9^ ^)^ However, the factors contributing to the loss of fixation in diabetics are unclear.

T2DM is characterized by hyperglycemia as a result of insulin resistance and is associated with increased risk for cardiovascular‐related and all‐cause mortality.^(^ ^10^ ^)^ Although considerable attention has been paid to the association between hyperglycemia and the risk for early TJR complications, such as periprosthetic joint infection^(^ ^11^ ^)^ or impaired wound healing,^(^ ^5^ ^)^ there is also a risk for late complications, including aseptic loosening.^(^ ^8^ ^)^ Aseptic loosening results from poor implant fixation within the host bone, which can be due to poor initial fixation or loss of fixation over time due to mechanical or biological factors.^(^ ^12^ ^)^ A variety of factors contribute to implant fixation, including osseointegration, or the direct contact between bone and the implant material, bone structure, and matrix composition.^(^ ^13^, ^14^ ^)^ Importantly, each of these parameters can be directly influenced by T2DM pathophysiology. For instance, bone remodeling rates appear to be suppressed in patients with T2DM^(^ ^15^ ^)^ and cortical porosity is elevated in patients with T2DM,^(^ ^16^ ^)^ particularly in those with a history of skeletal fractures.^(^ ^17^, ^18^ ^)^ Finally, the bone matrix in patients with T2DM tends to have higher mineralization and collagen cross‐linking compared with non‐diabetics (summarized by Lekkala and colleagues^(^ ^19^ ^)^). Clinically, T2DM impairs dental implant osseointegration,^(^ ^20^ ^)^ and animal studies have suggested that the same is true in rats receiving transcortical titanium screws.^(^ ^21^ ^)^ However, it is less clear whether T2DM impairs osseointegration in intramedullary implants, a model commonly used to study orthopedic implant integration.^(^ ^22^ ^)^

A previous study from our laboratory reported that osseointegration is the single most important factor in determining implant fixation strength in normoglycemic and diabetic rats receiving intramedullary implants.^(^ ^13^ ^)^ Despite the clear deleterious effects of T2DM on implant fixation, our previous study was not designed to examine the potential contributions of T2DM disease‐related factors, such as glycemic control, suppressed bone remodeling,^(^ ^23^, ^24^ ^)^ and systemic inflammation,^(^ ^25^ ^)^ to implant fixation. Further, our previous study utilized the Zucker Diabetic Fatty (ZDF) rat as a model of T2DM. The ZDF model harbors a leptin receptor mutation to induce hyperglycemia.^(^ ^26^ ^)^ Although this model recapitulates many T2DM disease characteristics, leptin receptor mutations are rare in humans.^(^ ^27^ ^)^ Therefore, the current study utilized the Zucker Diabetic‐Sprague Dawley (ZDSD) rat model of T2DM, a model that develops progressive hyperglycemia and obesity without leptin receptor mutations.^(^ ^28^ ^)^ We investigated the effects of T2DM on implant osseointegration, peri‐implant bone structure and matrix composition, and fixation strength by comparing ZDSD rats to Sprague Dawley controls. Additionally, we evaluated the relative contribution of glucose and cytokine levels, bone formation rates, structure, and matrix composition to both osseointegration and implant fixation strength. We hypothesized that, similar to our previous study in the ZDF model, T2DM would negatively affect osseointegration and that the extent of osseointegration and both peri‐implant bone structure and matrix composition would contribute to implant fixation strength.

## Materials and Methods

### Animals

All animal experiments were performed under institutionally approved protocols. Fifteen‐week‐old male outbred Sprague Dawley (SPD, *n* = 16) and Zucker Diabetic‐Sprague Dawley rats (*n* = 16) were purchased from Charles River (Wilmington, MA, USA). At 16 weeks of age, both SPD and ZDSD rats were provided a high‐fat diet (Research Diets Inc, New Brunswick, NJ, USA; #D12468, 28.8% kcal from fat) for 2 weeks to induce a type 2 diabetic phenotype in the ZDSD group, consistent with the supplier's recommendations (purchased from Charles River, but now sold through Crown Bioscience, San Diego, CA, USA) and previously published studies.^(^ ^29^ ^)^ An animal was considered diabetic after achieving two preoperative blood glucose measures >250 mg/dL. After diabetic status was confirmed in the ZDSD rats, all animals were switched to a standard rodent diet (Purina LabDiet, St. Louis, MO, USA; 5008, 6.5% kcal from fat) for the remainder of the experiment. All groups were allowed access to food and water *ad libitum*.

### Implants

Fifteen‐millimeter‐long by 1.5‐mm‐diameter titanium rods (99.6% in purity, Goodfellow, Oakdale, PA, USA) were roughened by sonicating in hexane, methanol, acetone, and water for 15 minutes each, followed by dual acid etching according to previous established methods.^(^ ^30^ ^)^ Implants were sterilized in 70% ethanol overnight. After drying at room temperature, the implants were kept in sterile saline until surgery to prevent oxidation.

### Rodent surgery

All animals had bilateral titanium implants placed into their distal femoral canals at 18 weeks of age. After implant placement, animals were then randomly divided into two groups for postsurgical euthanization: 2 weeks and 10 weeks. The postsurgical time points were selected based on our previous study with the ZDF rat,^(^ ^13^ ^)^ which showed increasing osseointegration and fixation strength in these two time points, and a study describing the longitudinal remodeling rates after intramedullary implant placement in the rat model that noted gradually increasing bone formation and resorption rates from implant placement to 8‐week postsurgery and a trend toward declining rates at 12‐week postsurgical placement.^(^ ^31^ ^)^ The anticipated sample size of 8 per group was selected based on prior experiments demonstrating significant differences in the fixation strength between Sprague Dawley and ZDF.^(^ ^13^ ^)^ Two animals in the SPD group were excluded because of poor placement of the titanium rods, resulting in a final sample size of 7 and 8 for SPD and ZDSD rats at each postsurgical euthanization time, respectively. At study termination, right femurs were removed, wrapped in saline‐soaked gauze, and frozen at −20°C until further processing was performed.

### Glucose monitoring

Nonfasted blood samples were taken weekly from the tail vein using a straight razor and measured using a Clarity BG1000 Blood Glucose Monitoring System (Clarity Diagnostics, Boca Raton, FL, USA). All glucose measures were taken in the afternoon (between 12 p.m. and 5 p.m.), except for measurements on the day of surgery, which were taken in the morning (between 8 a.m. and 12 p.m.).

### Fluorochrome labeling

All animals received dual subcutaneous fluorochrome injections before study termination. Calcein (25 mg/kg, Sigma, St. Louis, MO, USA) was injected 10 days before study termination, and oxytetracycline hydrochloride (25 mg/kg, Sigma) was injected 2 days before study termination.

### Blood sampling

Tail vein blood samples were taken from all rats 2 days postoperatively. Blood samples were allowed to clot at room temperature for 30 minutes before being centrifuged at 3400*g* for 15 minutes at 4°C. The resulting sera samples were separated and aliquoted for subsequent measurements.

### Circulating bone turnover markers and cytokines

Circulating C‐terminal telopeptide of type I collagen (CTX‐1) and N‐terminal propeptide of type I procollagen (P1NP) were measured using commercially available ELISA assays (Immunodiagnostic Systems, Boldon, UK: CTX intra‐assay coefficient of variation [CV] = 13%/interassay CV = 11% and P1NP intra‐assay CV = 10.1%/interassay CV = 8.4%). Circulating monocyte chemoattractant protein‐1 (MCP‐1) and interleukin‐10 (IL‐10) were evaluated using a Luminex Multiplex assay (EMD Millipore Corporation, Burlington, MA, USA; RECYTAMG‐65 K, intra‐assay CV < 10%). Some samples were below the lower limits of detection for IL‐10, and the concentration values were set at one‐half this concentration for the sake of statistical analysis (3.66 pg/mL). All kits were performed according to the manufacturer's recommended protocols using methods previously described.^(^ ^32^, ^33^ ^)^

### Slab preparation and selection

**Fig. 1:**
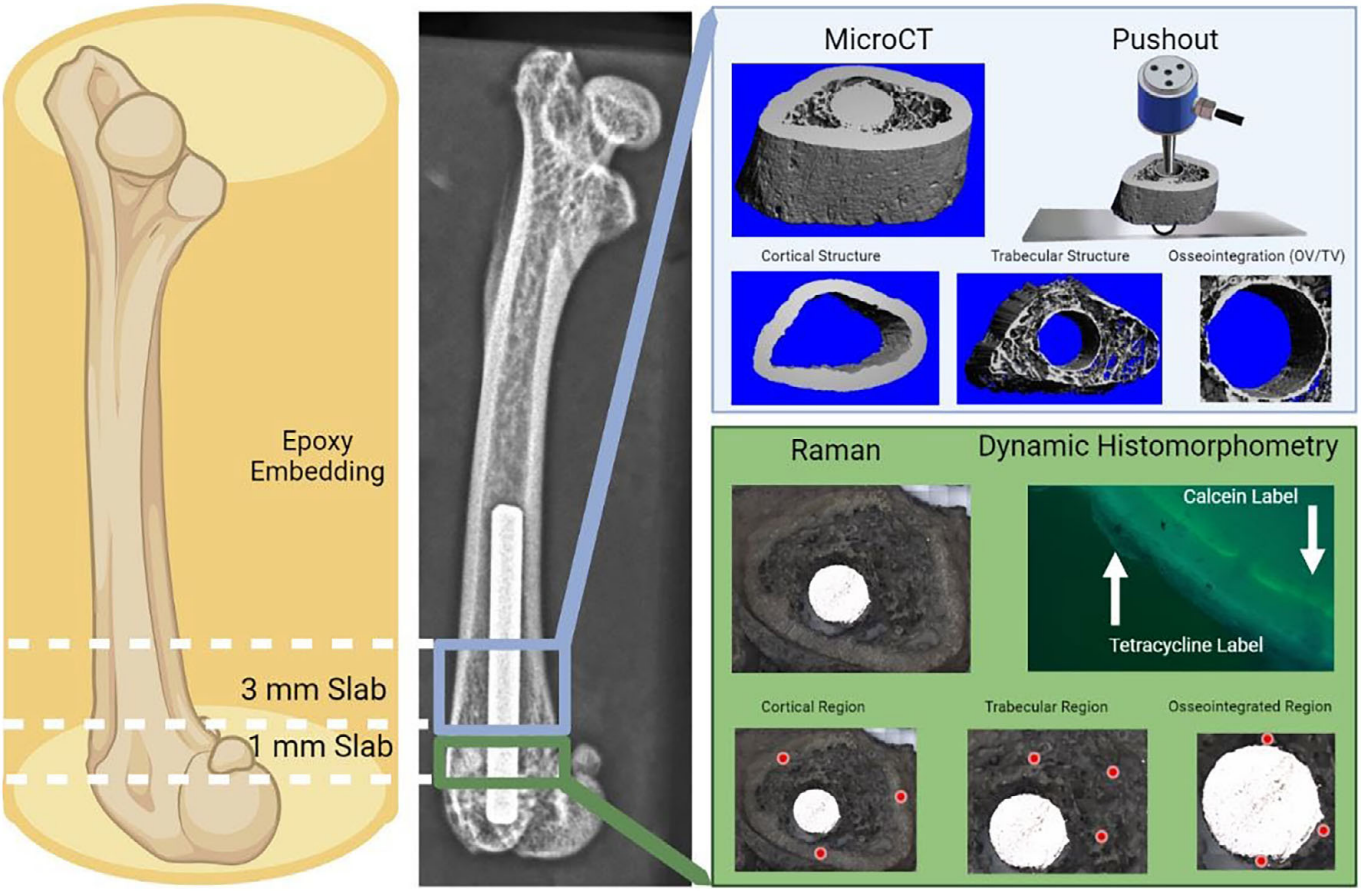
Flowchart of the sample handling process. Fresh frozen femurs were encased in noninfiltrating epoxy and sectioned. One 1‐mm slab just proximal to the distal growth plate was used to assess matrix composition (Raman) and bone formation (dynamic histomorphometric) measurements. An approximate representation of the Raman spectra collection sites are presented in the cortical, trabecular, and osseointegrated regions. The adjacent 3‐mm slab was used to assess bone structure (micro‐CT) and interfacil strength (pushout testing).

Harvested femora were encased in a non‐infiltrating epoxy resin (EpoThin, Buehler, Lake Bluff, IL, USA) and transversely cut starting from the distal end, proximally into ~1‐mm‐thick slabs (Exakt, Oklahoma City, OK, USA) until the distal growth plate was reached. The first ~1‐mm slab to have a proximal surface free of growth plate was used for dynamic histomorphometry and Raman spectroscopic analysis (described below). A second slab was generated by cutting ~3 mm proximal to the slab used for histomorphometry and Raman and was used for microcomputed tomography and mechanical pushout testing. A graphical representation of the slide preparation process is presented in Figure 1. Both slabs were then hand‐ground to provide a smoothened surface and to remove burrs on the titanium implant surface created during cutting. After grinding, the epoxy embedding material was removed by gently cutting away the epoxy resin. Slabs were subsequently frozen before further analysis and all samples underwent one freeze/thaw cycle before mechanical pushout testing.

### Dynamic histomorphometry

To investigate bone remodeling, we performed dynamic histomorphometric analysis on undecalcified bone at the endocortical (Ec), trabecular (Tb), and osseointegrated (Os) bone surfaces (Osteomeasure, Decatur, GA, USA). The mineral apposition rate (MAR) was assessed between calcein and tetracycline labels. Mineralizing surface per bone surface (MS/BS) was determined as the amount of double‐labeled perimeter plus one half of the single‐label perimeter divided by bone surface perimeter and multiplied by 100. The bone formation rate (BFR/BS) was calculated by multiplying the MAR by MS/BS. When no double label was detected, an imputed value of 0.3 μm/d was used in place of zero to allow for calculation of BFR/BS.

### Micro‐computed tomography (microCT)

Three‐millimeter slabs were submerged in PBS to prevent dehydration and imaged with micro‐CT under two distinct imaging protocols. The first protocol measurement peri‐implant bone structure along the entire 3‐mm slab length (Scanco [Bruttisellen, Switzerland] μCT50 14.8 μm isotropic voxels, 90 kVp, 88 μA, 750 ms integration, 0.5 mm Al filter). Variables determined using the first protocol included: trabecular bone volume fraction (BV/TV), trabecular number (Tb.N), trabecular thickness (Tb.Th), trabecular spacing (Tb.Sp), cortical bone area (Ct.Ar), total cortical area (Tt.Ar), cortical porosity (Ct.Po), and cortical thickness (Ct.Th). The second protocol measured the osseointegration volume per total volume (OV/TV) by scanning a 1.5‐mm region of interest set at 50% of the slab thickness (Scanco μCT50 1.5 μm isotropic voxels, 90 kVp, 88 μA, 750 ms integration, 1600 projections/180°, 0.5 mm Al filter).

### Raman spectroscopy

#### Region selection

Bone matrix composition was assessed in three compartments: endocortical (Ec), trabecular (Tb), and osseointegrated (Os), the latter being bone within 50 μm of the implant surface. Three spectra were collected and averaged to obtain a single matrix compositional measurement within each compartment per animal. Spectra from cortical bone were collected approximately halfway between the endosteal and periosteal surface. Spectra from trabecular bone was collected at approximately half the thickness of individual trabeculae.

#### Spectral acquisition and analysis

All Raman spectra were collected using a 785 nm, 100 mW laser, a 600 gr/mm grating, and a 50× objective with a 12‐second acquisition time (Horiba [Kyoto, Japan] LabRAM HR Evolution). The resulting spectra were evaluated according to previously defined matrix composition spectral peaks^(^ ^34^, ^35^ ^)^ and integrated areas calculated by PLS Toolbox (version 8.9; Eigenvector Research Inc., Manson, WA, USA). The mineral‐to‐matrix ratio was defined using both the ν1 and ν2 phosphate peaks. Importantly, the ν1 peak is more dependent on the orientation of the hydroxyapatite crystals, whereas the ν2 is less affected by changes in mineral orientation.^(^ ^36^ ^)^ Specifically, the ratio of the integrated areas of _ν1_PO_4_ at ~960 cm^−1^ or _ν2_PO_4_ at ~427 cm^−1^ per proline at ~855 cm^−1^ were used. Type B carbonate substitution was defined by the ratio of the integrated areas of the CO_3_ peak at ~1072 cm^−1^ per either the _ν1_PO_4_ or _ν2_PO_4_ peaks. The hydroxyproline/proline ratio was used as an indication of hydroxylation of proline, which has been reported in response to glycation^(^ ^37^ ^)^ (Hyp at ~877 cm^−1^ per Pro at ~922 cm^−1^). Carboxy‐methyl‐lysine content was defined as the peak at ~1150 cm^−1^ per CH_2_‐wag (~1450 cm^−1^). The full width at half maximum of the _ν1_PO_4_ peak (at ~960 cm^−1^) was calculated by Origin (version 2019; OriginLab, Northampton, MA, USA) and defined as a measure of crystallinity. The outcome variables include mineral‐to‐matrix ratio (mineral‐to‐matrix), type B carbonate substitution (carb. sub.), post translational modifications (post trans. mod.), carboxy‐methyl‐lysine content (CML content), and crystallinity.

#### Pushout testing

Implant fixation strength was assessed within the same slab samples used for the μCT analysis. Slabs were thawed and tested sequentially to maintain consistency of temperature when performing the mechanical test. Pushout testing was performed using the Criterion 43 (MTS Systems, Eden Prairie, MN, USA) according to previously established protocol^(^ ^38^ ^)^ using a 304 stainless steel dowel pin (UXCell, Hong Kong) with dimensions of 15.8 mm × 1 mm as the pushing rod and a stainless steel base with a 2.5‐mm hole. The sample was centered over the hole in the base and preloaded with 1 N. Tests were conducted at 0.1 mm/s displacement and 100 Hz data acquisition. The resultant force‐displacement curves were used to calculate the force at failure. Implant fixation strength was calculated by dividing this force by the implant surface area, which was calculated according to the surface area of a 1.5‐mm by 3‐mm cylinder.

### Statistical analysis

Prism (version 9; GraphPad, San Diego, CA, USA) and SPSS (version 26.0; IBM Corp., Armonk, NY, USA) software packages were used for plotting and data analysis, respectively. Longitudinally measured body weight and blood glucose levels were compared using a repeated two‐way analysis of variance (ANOVA) to determine the effects of group (SP versus ZDSD), time (2 versus 10 weeks post‐implant placement), and the group‐by‐time interaction, with a significance threshold of *p* < 0.05. Blood samples collected 2 days after surgery were compared between groups using an independent samples *t* test. For study endpoints evaluated after euthanasia, including circulating biomarkers, microCT‐based structural parameters, Raman‐based matrix composition, dynamic histomorphometric measures, and implant fixation, a standard two‐way analysis of variance was used to determine the effects of group, time, and the group‐by‐time interaction. When any of the group effects were significant, a Student's *t* test was used to determine post hoc significance, with an adjusted significance threshold of *p* < 0.025 (adjusted for multiple comparisons using a Bonferroni correction).

Stepwise linear regression was used to determine the factors that contribute to osseointegration volume per total volume (OV/TV) and implant fixation strength. Potential predictors of both OV/TV and fixation strength that were entered into the model are presented in Table 1. Similar models were run, including the time to euthanasia, but this did not affect the outputs and therefore was not included in the analysis.

**Table 1:** Stepwise Linear Regression Model Parameters

Dependent variable	Independent variables evaluated
Osseointegrated volume per total volume (OV/TV)	Bone remodeling
	Tb.MS/BS, Tb.MAR, Tb.BFR/BS
	Ec.MS/BS, Ec.MAR, Ec.BFR/BS
	Os.MS/BS, Os.MAR, Os.BFR/BS
	Glucose
	Week 0 glucose, week 1 glucose
	Inflammation
	Post‐op MCP‐1, Post‐op IL‐10
Fixation strength	Bone remodeling
	Tb.MS/BS, Tb.MAR, Tb.BFR/BS
	Ec.MS/BS, Ec.MAR, Ec.BFR/BS
	Os.MS/BS, Os.MAR, Os.BFR/BS
	Glucose
	Week 0 glucose, week 1 glucose
	Inflammation
	Post‐op MCP‐1, Post‐op IL‐10
	Bone structure
	BV/TV, Tb.N, Tb.Th, Tb.Sp, Ct.Ar, Tt.Ar, Ct.Po, Ct.Th, OV/TV
	Matrix composition^a^
	Cortical mineral‐to‐matrix, cortical carbonate substitution, cortical crystallinity cortical posttranslational modifications, cortical CML content
	Trabecular mineral‐to‐matrix, trabecular carbonate substitution, trabecular crystallinity trabecular posttranslational modifications, trabecular carboxy‐methyl‐lysine (CML) content
	Osseointegrated mineral‐to‐matrix, trabecular carbonate substitution, trabecular crystallinity trabecular posttranslational modifications, trabecular CML content

*Note*: Post‐op refers to tail vein blood samples collected at 2 days after surgery.

^a^ Both mineral‐to‐matrix and carbonate substitution ratios based on the _ν1_PO_4_ and _ν2_PO_4_ peak were included.

Although not initially part of the hypothesis or study design, we noted unexpected variability in the glycemia status of the ZDSD animals. Specifically, 10 ZDSD animals remained above the 250 mg/dL diabetic threshold throughout the study timeline, and 6 animals had measurements that fluctuated above and below the diabetic threshold for rats. Of the 6 animals with variable hyperglycemia, 3 animals terminated at week 2 and 3 animals terminated at week 10. These rats had between 2 and 3 glucose measurements that dropped below the 250 mg/dL threshold. Therefore, to evaluate the effect of glycemic status on our primary endpoints, the ZDSD rats were further stratified into two groups, sustained or variable diabetic status, and two‐way ANOVAs were rerun with three groups: SPD, ZDSD sustained, and ZDSD variable hyperglycemia. As the study was not powered to determine the effects of variable versus sustained hyperglycemia, we kept a significance threshold of *p* < 0.05 for both ANOVA and post hoc comparisons.

## Results

### Longitudinal glucose levels and body weight

**Fig. 2:**
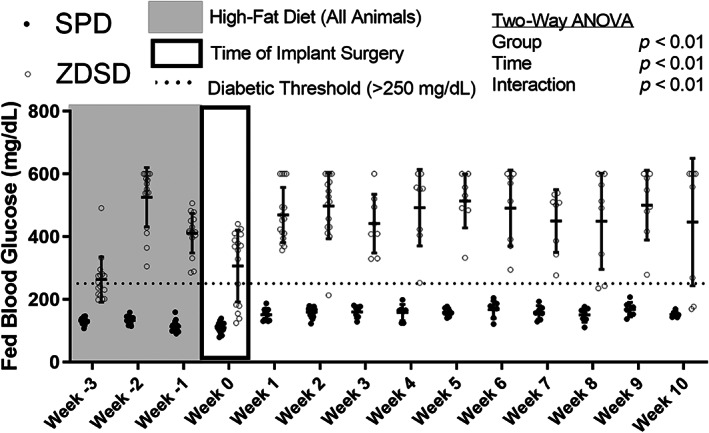
Blood glucose levels in Sprague Dawley (SPD) and Zucker Diabetic‐Sprague Dawley (ZDSD) groups. Data are reported as the means and standard deviations with each data point representing an individual animal. Week −3 through week 10 represent the experimental timeline with week 0 representing the time of surgery. The shaded area represents the period at which both groups received a high‐fat diet. Results from a repeated two‐way analysis of variance are reported in the legend. Although not presented for simplicity, the post hoc analysis found that ZDSD rats had significantly higher glucose levels at each time point compared with SPD rats.

Non‐fasted blood glucose was measured longitudinally using a glucometer. Blood glucose was significantly altered by group, time, and the group‐by‐time interaction (Fig. 2). ZDSD rats drastically increased blood glucose levels during the 3 weeks of high‐fat diet, with all animals exceeding the 250 mg/dL diabetic threshold by week 3. Most ZDSD rats maintained persistent hyperglycemia after return to standard chow, although some dropped below the 250 mg/dL threshold over the course of the study. SPD animals did not increase glucose levels during the high‐fat diet period and remained below the 250 mg/dL diabetic threshold throughout the course of the study.

Body weight was significantly altered by group, time, and the group‐by‐time interaction (Supplemental Fig. S1). Overall, the ZDSD rats had elevated body weight compared with the SPD rats. Both SPD and ZDSD rats had a slight body weight increase during the period of high‐fat feeding. Specifically, SPD rats gained 54 g, or 13.9%, of their body weight and ZDSD rats gained 41 g, or 8.3%, of their body weight over the high‐fat diet feeding period. Two weeks after cessation of the high‐fat diet, SPD rats lost 10 g, or 2.2%, and ZDSD rats lost 39 g, or 7.3%, of their body weight.

### Bone formation

#### Endocortical bone formation

**Fig. 3:**
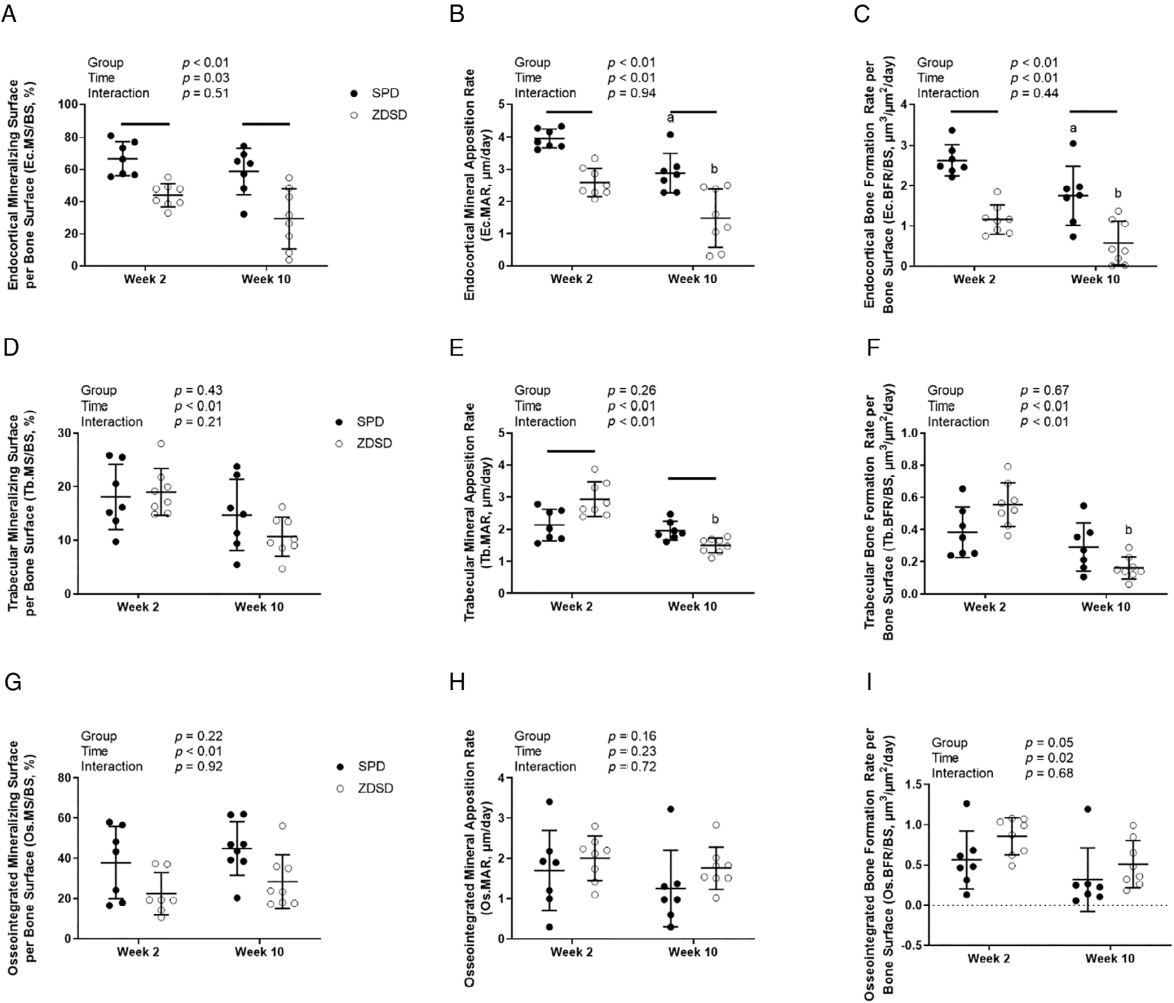
Bone formation measured at the endocortical (*A–C*) and trabecular (*D–F*) compartments and in bone in direct contact with the implant (*G–I*). (*A, D, G*) Mineralizing surface per bone surface (MS/BS), (*B, E, H*) mineral apposition rate (MAR), and (*C, F, I*) bone formation rate per bone surface (BFR/BS) are presented for Sprague Dawley (SPD) and Zucker Diabetic‐Sprague Dawley (ZDSD) groups. Data are reported as the means and standard deviations with each data point representing an individual animal. Week 2 and week 10 represent time elapsed since the implant surgery. Results from a two‐way analysis of variance are reported in the legend. Bars above the data indicate significant differences between groups at the same time point, whereas differences between SPD at weeks 2 and 10 are indicated with an “a” and between ZDSD at weeks 2 and 10 with a “b” above the data.

Dynamic histomorphometry was used to assess bone formation rates at three separate compartments: endocortical, trabecular, and osseointegrated, or bone in direct contact with the implant surface. Endocortical mineralizing surface per bone surface (Ec.MS/BS) was significantly influenced by group and time but not the group‐by‐time interaction (Fig. 3*A*). The Ec.MS/BS was depressed in ZDSD rats compared with SPD rats at both weeks 2 and 10, and there was a general decrease in Ec.MS/BS over time in both groups. Similarly, both the endocortical mineral apposition rate (Ec.MAR) and endocortical bone formation rate per bone surface (Ec.BFR/BS) were influenced by group and time but not by the group‐by‐time interaction (Fig. 3*B*, *C*). ZDSD rats had decreased Ec.MAR and Ec.BFR/BS compared with the SPD rats at both weeks 2 and 10. Similar to the Ec.MS/BS, the Ec.MAR and Ec.BFR/BS in both groups decreased with time.

#### Trabecular bone formation

Tb.MS/BS was not significantly influenced by group or the group‐by‐time interaction but did have a significant time effect (Fig. 3*D*). Specifically, Tb.MS/BS declined in both SPD and ZDSD groups over time. The Tb.MAR was not affected by group but was influenced by both time and the group‐by‐time interaction (Fig. 3*E*). The ZDSD rats had significantly higher Tb.MAR at week 2, but significantly lower levels at week 10, compared with SPD rats. Tb.BFR/BS was significantly affected by the time and group‐by‐time interaction but not by group (Fig. 2*F*). Tb.BFR/BS showed a similar trend, but the post hoc comparisons failed to meet the adjusted *p* value threshold.

#### Osseointegrated bone formation

Osseointegrated MS/BS (Os.MS/BS) was not influenced by group or the group‐by‐time interaction but was significantly affected by time (Fig. 3*G*). Specifically, Os.MS/BS in both SPD and ZDSD rats tended to increase with time. Os.MAR was not affected by group, time, or the group‐by‐time interaction (Fig. 3*H*). Os.BFR/BS was influenced by both group and time but not the group‐by‐time interaction (Fig. 3*I*). Overall, ZDSD rats had higher Os.BFR/BS, but the specific post hoc comparisons at each time point were not significant. The Os.BFR/BS decreased in both SPD and ZDSD rats over time.

#### Circulating bone turnover markers

**Fig. 4:**
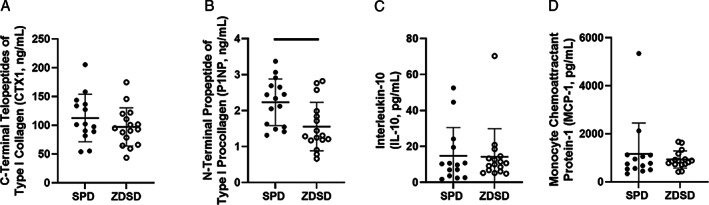
Circulating bone turnover markers and inflammatory cytokines collected 2 days post‐implant placement. (*A*) CTX‐1, (*B*) P1NP, (*C*) IL‐10, and (*D*) MCP‐1 measured in Sprague Dawley (SPD) and Zucker Diabetic‐Sprague Dawley (ZDSD) rats. Data are reported as the means and standard deviations with each data point representing an individual animal. Significant between‐group comparisons are presented as a bar over the data.

Circulating bone turnover markers were measured using commercial ELISAs on serum obtained via tail vein blood draw 2 days post‐implant placement surgery. There was no difference in CTX1 levels between SPD and ZDSD rats (Fig. 4*A*). Circulating P1NP was significantly decreased in the ZDSD rats compared with the SPD group (Fig. 4*B*). In the measurements made post euthanasia, there was a significant time effect for CTX1, with both SPD and ZDSD rats showing decreasing CTX1 over time (Supplemental Fig. S2*A*). However, there was no group or group‐by‐time interaction. There were also no significant effects for P1NP (Supplemental Fig. S2*B*).

### Circulating markers of inflammation

There were no significant effects detected in the postoperative circulating levels of IL‐10 or MCP‐1 measured 2 days after implant placement (Fig. 4*C*, *D*). There were also no statistically significant effects in IL‐10 or MPC‐1 in the measurements made at weeks 2 and 10 (Supplemental Fig. S2*C*, *D*).

### Osseointegration

**Fig. 5:**
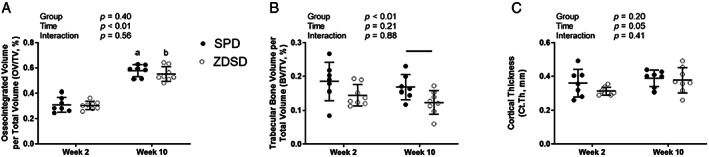
Osseointegrated volume per total volume (OV/TV) and bone structure in Sprague Dawley (SPD) and Zucker Diabetic‐Sprague Dawley (ZDSD) rats. (*A*) OV/TV, (*B*) trabecular bone volume per total volume (BV/TV), (*C*) cortical thickness (Ct.Th). Data are reported as the means and standard deviations with each data point representing an individual animal. Week 2 and week 10 represent time post‐implant placement surgery. Results from a two‐way analysis of variance are reported in the legend. Bars above the data indicate significant differences between groups at the same time point, whereas differences between SPD at weeks 2 and 10 are indicated with an “a” and between ZDSD at weeks 2 and 10 with a “b” above the data.

Osseointegrated volume per total volume (OV/TV) was obtained using high‐resolution micro‐CT. OV/TV was not significantly altered by group (Fig. 5*A*). OV/TV increased with time in both SPD and ZDSD groups as denoted by the significant time effect and the lack of a significant group‐by‐time interaction.

### Bone microarchitecture

Bone microarchitecture was assessed in the peri‐implant region using micro‐CT. Trabecular bone volume per total volume (BV/TV) was significantly influenced by group (Fig. 5*B*). Although there were no significant group differences at week 2, at week 10 ZDSD rats had significantly reduced trabecular BV/TV compared with SPD rats. Cortical thickness (Ct.Th) was not influenced by group but did have a significant time effect denoted by a slight increase in Ct.Th over time (Fig. 5*C*).

Within the cortical compartment, the cortical porosity was significantly influenced by time, with a general trend toward decreased cortical porosity in both groups (Table 2). No additional significant effects were noted in the cortical bone. In the trabecular compartment, trabecular number was significantly influenced by group and time but not the group‐by‐time interaction. Trabecular number was lower in the ZDSD rats compared with the SPD, and both groups had decreasing trabecular number with time. The trabecular thickness had a significant group‐by‐time interaction, with ZDSD rats having greater trabecular thickness at week 2 post‐implant placement compared with SPD rats but no difference at week 10. Trabecular spacing was significantly influenced by group and time. Trabecular spacing was greater in the ZDSD rats compared with the SPD rats at week 2 and both groups increased trabecular spacing over time. Finally, the connectivity density was affected by group and time, with ZDSD rats having decreased connectivity density compared with SPD rats at both 2 and 10 weeks post‐implant placement.

**Table 2:** Micro‐CT‐Derived Bone Microarchitecture Parameters

	Week 2		Week 10		Two‐way ANOVA results		
Group	SPD	ZDSD	SPD	ZDSD	Group	Time	Interaction
Bone area (mm^2^)	5.90 (0.49)	6.27 (0.11)	5.97 (0.78)	6.29 (0.89)	0.15	0.84	0.90
Total area (mm^2^)	6.21 (0.65)	6.66 (0.15)	6.10 (0.82)	6.43 (0.82)	0.12	0.51	0.81
Cortical porosity (%)	4.84 (3.71)	5.74 (1.47)	2.08 (1.60)	2.35 (2.31)	0.51	**<0.01**	0.72
Trabecular number (––)	3.53 (0.70)	2.46 (0.35)	**2.30 (0.77)** ^**a**^	**1.89 (0.35)** ^**b**^	**0.01**	**<0.01**	0.12
Trabecular thickness (mm)	0.076 (0.016)	0.087* (0.006)	**0.092 (0.011)** ^**a**^	**0.084 (0.010)** ^**b**^	0.67	0.11	**0.03**
Trabecular spacing (mm)	0.29 (0.47)	0.42* (0.06)	0.47 (0.14)	0.55 (0.10)	**0.01**	**<0.01**	0.48
Connectivity density (––)	85.65 (30.27)	45.29 (9.92)*	42.45 (15.07)	25.93 (11.02)*	**<0.01**	**<0.01**	0.08

*Note*: Data are presented as the mean (standard deviation). Results from the two‐way ANOVA are presented on the right. Significant post hoc differences between groups at each time point are indicated with a bolded*. Significant post hoc differences between Sprague Dawley (SPD) rats between weeks 2 and 10 are indicated with a bold “a” and between Zucker Diabetic‐Sprague Dawley (ZDSD) rats with a bolded “b.”

### Bone matrix composition

Bone matrix composition was measured in three separate compartments—cortical, trabecular, and bone in direct contact with the implant (osseointegrated bone)—using Raman microspectroscopy. Within the cortical compartment, the only significant effect was a time effect for carbonate substitution. There was an overall trend toward decreasing cortical carbonate substitution over time in both groups (Table 3). In the trabecular compartment, the only significant effect was a time effect for the mineral‐to‐matrix ratio. Both SPD and ZDSD groups tended toward an increase in the trabecular mineral‐to‐matrix ratio over time. The osseointegrated mineral‐to‐matrix was significantly affected by group. Specifically, ZDSD rats had decreased mineral‐to‐matrix when compared with SPD at 10 weeks post‐implant placement. The osseointegrated carbonate substitution had a significant group‐by‐time interaction due to a decrease in carbonate substitution over time in SPD rats and an increase over time in the ZDSD rats. Finally, there was a significant group effect for the osseointegrated crystallinity parameter. ZDSD rats had greater crystallinity at week 10 compared with SPD rats.

**Table 3:** Raman Microspectroscopy‐Derived Bone Matrix Composition

	Week	2	Week	10	Two‐way	ANOVA	Results
Group	SPD	ZDSD	SPD	ZDSD	Group	Time	Interaction
Cortical matrix composition							
Mineral‐to‐Matrix (_ν1_PO_4_)	32.63 (10.15)	29.90 (11.99)	35.34 (10.13)	30.55 (9.96)	0.34	0.67	0.79
Mineral‐to‐Matrix (_ν2_PO_4_)	0.97 (.38)	1.37 (1.01)	1.20 (0.43)	1.80 (0.71)	0.06	0.21	0.72
Carb. Sub. (_ν1_PO_4_)	0.33 (0.07)	0.32 (0.05)	**0.25 (0.02)** ^**a**^	0.31 (0.03)*	0.15	**0.03**	0.06
Carb. Sub. (_ν2_PO_4_)	5.04 (1.29)	5.19 (2.13)	4.26 (0.93)	4.99 (1.51)	0.44	0.40	0.62
Post Trans. Mod.	1.17 (0.43)	1.09 (0.79)	0.96 (0.27)	1.22 (0.25)	0.63	0.82	0.36
CML Content	0.05 (0.02)	0.05 (0.02)	0.05 (0.03)	0.04 (0.01)	0.98	0.43	0.32
Crystallinity	17.50 (0.66)	17.48 (0.78)	17.35 (0.87)	17.46 (0.47)	0.86	0.74	0.79
Trabecular matrix composition							
Mineral‐to‐Matrix (_ν1_PO_4_)	40.56 (24.91)	39.55 (30.35)	59.43 (36.37)	33.75 (12.86)	0.21	0.53	0.25
Mineral‐to‐Matrix (_ν2_PO_4_)	1.13 (0.50)	1.78 (2.08)	1.44 (0.67)	1.81 (1.06)	0.31	0.74	0.78
Carb. Sub. (_ν1_PO_4_)	0.29 (0.05)	0.30 (0.05)	0.28 (0.05)	0.32 (0.05)	0.20	0.67	0.56
Carb. Sub. (_ν2_PO_4_)	7.94 (9.00)	4.68 (1.48)	4.28 (1.99)	5.50 (1.86)	0.58	0.44	0.23
Post Trans. Mod.	1.16 (0.89)	0.92 (0.50)	1.02 (0.05)	1.07 (0.36)	0.66	0.97	0.49
CML Content	0.06 (0.02)	0.06 (0.03)	0.06 (0.03)	0.06 (0.03)	0.90	0.85	0.77
Crystallinity	18.51 (0.75)	18.42 (0.53)	18.38 (0.53)	18.47 (0.72)	0.98	0.87	0.73
Osseointegrated bone matrix composition							
Mineral‐to‐Matrix (_ν1_PO_4_)	37.13 (17.94)	30.15 (14.05)	38.19 (8.82)	22.97* (10.82)	**0.03**	0.54	0.42
Mineral‐to‐Matrix (_ν2_PO_4_)	0.87 (0.55)	1.87 (2.23)	1.26 (0.74)	1.59 (0.62)	0.18	0.91	0.50
Carb. Sub. (_ν1_PO_4_)	0.29 (0.07)	0.27 (0.03)	0.25 (0.04)	0.35 (0.09)*	0.07	0.34	**0.01**
Carb. Sub. (_ν2_PO_4_)	4.88 (0.97)	4.65 (1.25)	4.85 (1.18)	5.39 (1.65)	0.75	0.47	0.43
Post Trans. Mod.	1.09 (0.71)	1.03 (0.36)	1.04 (0.29)	1.19 (0.83)	0.63	0.80	0.63
CML Content	0.05 (0.01)	0.08 (0.02)*	0.03 (0.02)	0.15 (0.25)	0.12	0.55	0.35
Crystallinity	18.56 (0.65)	19.26 (1.27)	17.91 (0.71)	19.45* (1.20)	**0.01**	0.55	0.27

*Note*: Data are presented as the mean (standard deviation). Results from the two‐way ANOVA are presented on the right. Significant post hoc differences between groups at each time point are indicated with a bolded*. Significant post‐hoc differences between Sprague Dawley (SPD) rats between weeks 2 and 10 are indicated with a bold “a.” There were no differences between Zucker Diabetic‐Sprague Dawley (ZDSD) rats at weeks 2 and 10.

### Implant fixation strength

**Fig. 6:**
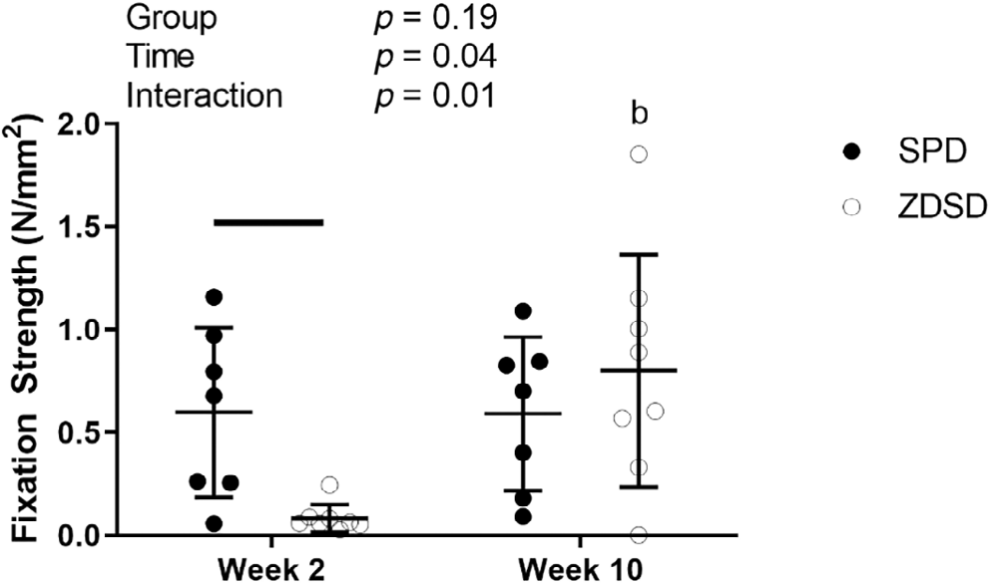
Implant fixation strength in Sprague Dawley (SPD) and Zucker Diabetic‐Sprague Dawley (ZDSD) rats. Data are reported as the means and standard deviations with each data point representing an individual animal. Week 2 and week 10 represent time post‐implant placement surgery. Results from a two‐way analysis of variance are reported in the legend. Bars above the data indicate significant differences between groups at the same time point. There were no differences between SPD at weeks 2 and 10, whereas differences between ZDSD at weeks 2 and 10 are indicated with a “b” above the data.

Implant fixation strength was measured using static pushout testing. Implant fixation strength was not significantly altered by group but did have significant time and group‐by‐time interaction effects (Fig. 6). ZDSD rats had significantly reduced fixation strength 2 weeks post‐implant placement, but the results were no longer significant at week 10.

### Predictors of OV/TV

A stepwise linear regression model was performed to determine the relative contributions of glycemic control, bone formation, and systemic inflammation to osseointegration volume/total volume (Table 4; see Table 1 for factors included in the model). The most significant variable was the Tb.MAR, explaining ~43.4% of the variance, followed by the circulating levels of MCP‐1 measured 1 week after surgery, which explained an additional 12.2% of the variance. In total, these two variables were able to account for 55.6% of the total variance in osseointegration volume/total volume.

**Table 4:** Factors That Contribute to Osseointegration Volume per Total Volume (OV/TV) in Sprague Dawley (SPD) and Zucker Diabetic‐Sprague Dawley (ZDSD) Rats

Stepwise model summary	*r* ^2^	Δ *r* ^2^	Adjusted *r* ^2^	Δ adjusted *r* ^2^	*p* Value
1. Tb.MAR	0.453		0.434		<0.001
2. Tb.MAR, MCP‐1 week 1	0.587	0.134	0.556	0.122	<0.001

### Predictors of implant fixation strength

A stepwise linear regression model was performed to determine the relative contributions of bone microarchitecture and bone matrix composition on implant fixation strength (Table 5). The most significant variable was Ct.Th, which explained 31.0% of the variance. Additional factors that independently contributed to implant fixation included the osseointegrated MAR (an additional 14.2%), the cortical carboxy‐methyl‐lysine content (CML, an additional 10.0%), the osseointegrated CML (an additional 8.6%), and the osseointegrated mineral‐to‐matrix ratio (an additional 5.4%). Overall, the combination of all five factors explained 69.2% of the variance in implant fixation strength.

**Table 5:** Factors That Contribute to Implant Fixation in Sprague Dawley (SPD) and Zucker Diabetic‐Sprague Dawley (ZDSD) Rats

Stepwise model summary	*r* ^2^	Δ *r* ^2^	Adjusted *r* ^2^	Δ adjusted *r* ^2^	*p* Value
1. Ct.Th	0.338		0.310		0.002
2. Ct.Th, Os.MAR	0.496	0.158	0.452	0.142	0.001
3. Ct.Th, Os.MAR, Cortical CML	0.606	0.110	0.552	0.100	0.001
4. Ct.Th, Os.MAR, Cortical CML, Osseointegrated CML	0.696	0.090	0.638	0.086	0.001
5. Ct.Th, Os.MAR, Cortical CML, Osseointegrated CML, Osseointegrated mineral‐to‐matrix (_v1_PO_4_)	0.754	0.058	0.692	0.054	0.001

### Effects of sustained and variable hyperglycemia

Interestingly, only 10 of the 16 ZDSD animals maintained a sustained hyperglycemia state, with glucose levels above 250 mg/dL, whereas 6 animals had variable hyperglycemia, with glucose levels intermittently dropping below 250 mg/dL over the course of the study. To determine whether the glycemic status of the animals influenced each of the measured endpoints, a separate analysis was run that separated the ZDSD group into sustained or variable hyperglycemia groups. A second set of analyses were run comparing three groups: SPD, sustained ZDSD, and variable ZDSD. The variables with statistically significant group (SPD versus sustained versus variable hyperglycemia) or group‐by‐time interactions are presented in Supplemental Tables S1 and S2. Bone formation, bone structure, and matrix composition variables were all affected by diabetic status. For example, fixation strength, Ec.MS/BS, trabecular thickness, and the osseointegrated carbonate substitution were all significantly affected by the group‐by‐time interaction, whereas Ec.MAR, BV/TV, and osseointegrated crystallinity were all affected by group (diabetic status). In general, animals with sustained hyperglycemia tended to have lower bone formation rates and decreased trabecular bone structural parameters when compared with the SPD or variable ZDSD groups (Supplemental Tables S1 and S2).

## Discussion

Orthopedic implant failure is a significant clinical challenge complicated by comorbidities such as type 2 diabetes. The current study used the Zucker Diabetic‐Sprague Dawley rat model to investigate the effects of T2DM on the establishment of osseointegration and implant fixation using an intramedullary implant model. The results demonstrate suppressed bone formation in the ZDSD rats, particularly in the endocortical compartment, and altered bone matrix composition. The extent of osseointegration was not affected by diabetes, whereas implant fixation was only transiently impaired. Bone formation in the peri‐implant trabecular bone and postoperative MCP‐1 levels contributed to variation in osseointegration, whereas cortical structure, bone formation around the implant, and matrix composition in both cortical and osseointegrated bone, specifically the carboxy‐methyl‐lysine content and mineral‐to‐matrix ratio, contributed to implant fixation strength. Contrary to our hypothesis and previous findings, osseointegration was not the primary contributing factor to implant fixation strength. Overall, the results further confirm the importance of bone‐quality parameters on orthopedic implant performance.

The skeletal complications of T2DM are multifaceted and include altered bone microstructure, remodeling, and matrix composition.^(^ ^39^, ^40^ ^)^ Similar to the findings from Creecy and colleagues,^(^ ^35^ ^)^ ZDSD rats in the current study had reduced trabecular bone volume per total volume and increased crystallinity levels when compared with SPD controls. In the current study, crystallinity was elevated primarily in the osseointegrated bone, which because of the timing of high‐fat diet feeding and implant placement, likely represents newly formed bone. Increasing crystallinity measured using the Raman technique is generally associated with an increase in crystal size or shape. It is notable that the reduction in mineral‐to‐matrix ratio within the osseointegrated bone tissue was found only when using the _v1_PO_4_ peak, which is highly dependent on mineral orientation.^(^ ^36^ ^)^ The mineral‐to‐matrix ratio calculated using the _v2_PO4 peak, which is more independent of mineral alignment, was not lower in ZDSD. Taken together, these results suggest that the mineral deposited in the ZDSD rats is more highly aligned but with altered shape or size. The mechanisms by which diabetes could influence bone matrix formation is not clear, but one potential explanation is the altered D‐spacing of collagen fibrils within both bones and tendons of ZDSD rats,^(^ ^29^ ^)^ which could impact the nucleation of hydroxyapatite crystals.

Hill Gallant and colleagues^(^ ^41^ ^)^ reported suppressed MS/BS and MAR, although the comparison in their study was between diabetic and non‐diabetic ZDSD rats. Although our study compared ZDSD to SPD, similar to their findings, we noted no difference in circulating CTX‐1 levels but suppressed tissue‐level bone formation. In our case, the results were limited to the endocortical surface, which is likely due to the proximity of the trabecular and osseointegrated compartments to the implant and the previously noted transient elevation in bone formation caused by the implant placement surgery.^(^ ^31^ ^)^ In further support of our findings, Hill Gallant and colleagues^(^ ^41^ ^)^ noted that the ZDSD rats that became diabetic had lower MS/BS and MAR than the non‐diabetic ZDSD, which is similar to the effects noted in the present study, where animals with sustained hyperglycemia had lower endocortical bone formation parameters than those with variable hyperglycemia.

Clinically, loss of implant fixation is one of the most common causes of total joint replacement failure.^(^ ^42^, ^43^ ^)^ The loss of implant fixation generally occurs by either failure to establish early osseointegration^(^ ^44^ ^)^ or late‐stage loss of fixation, generally due to aseptic loosening.^(^ ^42^ ^)^ Several preclinical studies have reported that animal models of T2DM have impaired osseointegration,^(^ ^45^, ^46^ ^)^ resulting in poor fixation between host bone and implant surface.^(^ ^21^, ^47^ ^)^ Although these previous studies have utilized a transcortical approach, or placement of the implant through the cortical bone,^(^ ^21^, ^45^, ^47^ ^)^ our study utilized an intramedullary approach to better simulate total joint replacement surgery. We have previously used this intramedullary rat model to evaluate the skeletal response to implant placement,^(^ ^31^ ^)^ the pathogenesis of particle‐induced loss of implant fixation strength,^(^ ^48^, ^49^ ^)^ and the efficacy of various anabolic treatments to promote implant osseointegration.^(^ ^50^, ^51^, ^52^ ^)^ Similar to our previous study using the Zucker Diabetic Fatty rat model of T2DM,^(^ ^13^ ^)^ we found no differences in osseointegration at 2 weeks post‐implantation. Unlike the ZDF model, the current study did not find a significant difference in osseointegration 10 weeks post‐implant placement. These differences may be because of the differences in animal strain (ZDF versus ZDSD). Although there are no studies comparing implant osseointegration in ZDF and ZDSD models, at least one study has measured bone structure and strength in the two models. Although the direct comparison is difficult because of the differing control strains, it should be noted that overall, both ZDF and ZDSD strains had reduced bone mass and strength compared with their controls.^(^ ^53^ ^)^ It is also possible that the differences between our current results and the earlier study was associated with the sex of the animals, as the previous study utilized female rats. To our knowledge, there have not been studies comparing osseointegration between male and female diabetic rats, but it is worth noting that estrogen, more specifically the loss of estrogen, is associated with increased implant failure.^(^ ^54^ ^)^

Surprisingly, the current study found that circulating MCP‐1, rather than glucose levels, was statistically associated with bone‐implant contact. Low‐grade inflammation is a hallmark of hyperglycemia.^(^ ^55^ ^)^ Although a variety of cytokines are affected by high glucose levels, we chose to focus on MCP‐1, which has been established as a regulator of glucose metabolism and insulin resistance,^(^ ^56^, ^57^ ^)^ diabetic wound healing,^(^ ^58^ ^)^ and as a potential genetic risk factor for T2DM (reviewed Panee^(^ ^59^ ^)^). Furthermore, high MCP‐1 levels have been reported in the synovial fluid of patients with failed joint arthroplasties.^(^ ^60^ ^)^ IL‐10 is reported to protect against high‐fat diet‐induced hyperglycemia^(^ ^61^, ^62^ ^)^ but also suppress the early phases of diabetic wound healing^(^ ^63^ ^)^ in mouse models of T2DM. Although IL‐10 appears involved in osteoarthritis disease progression,^(^ ^64^ ^)^ the role of IL‐10 in orthopedic implant failure is not well established. Therefore, it is perhaps not surprising that it did not contribute to either osseointegration or implant fixation strength. Because of the relatively low sera volume obtained from the tail vein blood draws, we did not pursue additional cytokine measurements. However, our lab has reported that early postoperative increases in interleukin‐6 (IL‐6) are associated with an increased risk for loss of fixation.^(^ ^65^ ^)^ Therefore, future work is needed to determine whether IL‐6, which also contributes to insulin resistance and hyperglycemia,^(^ ^66^ ^)^ contributes to implant related outcomes in T2DM.

The inconsistency in hyperglycemia after cessation of the high‐fat diet was unexpected. The two‐week high‐fat diet period was consistent with a previously published study that used a similar strategy to include hyperglycemia in male ZDSD animals^(^ ^29^ ^)^ and another that noted that ZDSD rats became diabetic (two consecutive measurements of hyperglycemia) after 2 weeks of high‐fat diet feeding.^(^ ^35^ ^)^ However, other studies have reported some variance in the onset of diabetes in the ZDSD strain. For example, Hill Gallant and colleagues^(^ ^41^ ^)^ reported that only 25% of the ZDSD rats developed diabetes with sustained high‐fat diet feeding. However, in this study, animals that became hyperglycemic maintained high glucose levels longitudinally throughout the study, while non‐diabetic rats did not cross the 250 mg/dL glucose threshold. It is worth noting that in our study, all ZDSD rats had at least two consecutive weekly glucose measurements above 250 mg/dL and therefore would fall into the classic diabetes classification for these models. The variance in hyperglycemia was only identified because we followed the glucose measurements weekly throughout the study duration. Although it is possible that the variance was related to measurement error, our exploratory analysis of sustained versus variance hyperglycemia and the differences noted therein tends to suggest that there is something inherently different about these animals. As we were not powered appropriately to test the contribution of variable hyperglycemia, we are unable to comment on the mechanism.

The strengths of the present study include investigating the potential contributions of three major disease factors to osseointegration and the independent effects of bone microarchitecture and matrix composition to implant fixation strength. Further, whereas other studies have investigated the effects of implant placement in diabetic rodents, this is the first implant study using the Zucker Diabetic‐Sprague Dawley rat model of diabetes, which has the advantage of not relying on mutant leptin receptor, as does the ZDF model,^(^ ^26^ ^)^ or use of toxic streptozotocin to induce diabetes.^(^ ^67^ ^)^ The limitations of this study include the wide variability in consistent diabetes in the ZDSD model, which likely increased the variability in some of the variables and masked group effects. However, this did present the unexpected advantage of investigating the effects of hyperglycemia. Additional technical limitations include the lack of tissue age–specific measurements of bone matrix composition and the limited number of individual spectra collected for each animal. Although we have used fluorescent labels in the past to make tissue age–specific measurements on rat tissues,^(^ ^68^ ^)^ the Raman instrument we used for this analysis was not equipped with fluorescent imaging. The lack of tissue‐age resolution could increase the intra‐specimen variability in the Raman measurements and prevents us from determining whether the measured matrix changes were due to differences in newly formed bone or changes to existing bone matrix. We attempted to limit the contribution of tissue age on the matrix composition measurements by collecting data within the center of boney structures, such as the middle of a given trabecula, but acknowledge that this does not fully avoid the confounding influence of matrix compositional dynamics. We only collected three individual spectra for each compartment, primarily to limit the collection time and due to the relatively limited tissue area available within the osseointegrated compartment. However, we acknowledge that this approach may underestimate heterogeneity of the bone matrix, which is shown to be suppressed in humans with type 2 diabetes.^(^ ^69^ ^)^ Finally, although rats are the most commonly used model to investigate the effects of T2DM on bone matrix, there have been discrepancies between some of the rat findings and data obtained from human tissues.^(^ ^19^ ^)^ So, although our data provide further evidence that deteriorated bone matrix composition contributes to mechanical changes in T2DM, further validation will be required in human tissues from TJR patients.

Our study demonstrates that T2DM negatively impacts peri‐implant bone formation, structure, and matrix composition. Surprisingly, the negative effects on the peri‐implant bone did not affect osseointegration and only transiently impaired fixation strength. The factors contributing to implant osteointegration included bone formation and systemic inflammation, while those contributing to implant fixation strength included bone structure, formation, and matrix composition, demonstrating the complexity of implant outcomes.

## Author Contributions

**Kyle D Anderson:** Conceptualization; formal analysis; investigation; writing – original draft; writing – review and editing. **Christian Beckmann:** Formal analysis; methodology; writing – review and editing. **Saskia Heermant:** Formal analysis; methodology; writing – review and editing. **Frank C Ko:** Conceptualization; writing – review and editing. **Bryan Dulion:** Formal analysis; investigation; writing – review and editing. **Imad Tarhoni:** Formal analysis; methodology; writing – review and editing. **Jeffrey A Borgia:** Investigation; methodology; writing – review and editing. **Amarjit S Virdi:** Investigation; methodology; writing – review and editing. **Markus A Wimmer:** Investigation; methodology; writing – review and editing. **D Rick Sumner:** Conceptualization; funding acquisition; writing – review and editing. **Ryan D Ross:** Conceptualization; formal analysis; funding acquisition; methodology; writing – original draft; writing – review and editing.

## Disclosures

The authors have no financial conflicts of interest or disclaimers to declare.

### Peer Review

The peer review history for this article is available at https://www.webofscience.com/api/gateway/wos/peer-review/10.1002/jbm4.10819.

## Supporting information


**Supplemental Fig. S1.** Body weight of Sprague Dawley (SPD) and Zucker Diabetic‐Sprague Dawley (ZDSD) groups. Data are reported as the means and standard deviations with each data point representing an individual animal. Week −3 through week 10 represent the experimental timeline with Week 0 representing the time of surgery. The shaded area represents the period at which both groups received high fat diet. Results from a repeated two‐way analysis of variance are reported in the legend. Post‐hoc differences are not presented within the figure, but ZDSD rats had higher body weight between week −3 and week 4. There were not differences between week 5 and week 10.
**Supplemental Fig. S2.** Circulating bone turnover markers and inflammatory cytokines collected at the time of euthanasia – 2‐ and 10‐weeks post implant placement. (A) CTX‐1, (B) P1NP, (C) IL‐10, and (D) MCP‐1 from Sprague Dawley (SPD) and Zucker Diabetic‐Sprague Dawley (ZDSD) groups. Data are reported as the means and standard deviations with each data point representing an individual animal. Results from a two‐way analysis of variance are reported in the legend. No post‐hoc differences were noted.
**Supplemental Table S1.** Variables Significantly Impacted by Diabetic Status
**Supplemental Table S2.** Post‐operative P1NP According to Diabetic Status
