# Supplementary material for: Zucker Diabetic‐Sprague Dawley Rats Have Impaired Peri‐Implant Bone Formation, Matrix Composition, and Implant Fixation Strength
Source: JBMR Plus. 2023 Oct 11;7(11):e10819. doi: 10.1002/jbm4.10819 (PMC10652173; doi:10.1002/jbm4.10819)
Supplement: Supplementary file 1 — Supplemental Fig. S1. Body weight of Sprague Dawley (SPD) and Zucker Diabetic‐Sprague Dawley (ZDSD) groups. Data are reported as the means and standard deviations with each data point representing an individual animal. Week −3 through week 10 represent the experimental timeline with Week 0 representing the time of surgery. The shaded area represents the period at which both groups received high fat diet. Results from a repeated two‐way analysis of variance are reported in the legend. Post‐hoc differences are not presented within the figure, but ZDSD rats had higher body weight between week −3 and week 4. There were not differences between week 5 and week 10. Supplemental Fig. S2. Circulating bone turnover markers and inflammatory cytokines collected at the time of euthanasia – 2‐ and 10‐weeks post implant placement. (A) CTX‐1, (B) P1NP, (C) IL‐10, and (D) MCP‐1 from Sprague Dawley (SPD) and Zucker Diabetic‐Sprague Dawley (ZDSD) groups. Data are reported as the means and standard deviations with each data point representing an individual animal. Results from a two‐way analysis of variance are reported in the legend. No post‐hoc differences were noted. Supplemental Table S1. Variables Significantly Impacted by Diabetic Status Supplemental Table S2. Post‐operative P1NP According to Diabetic Status [file JBM4-7-e10819-s001.docx]

**Zucker Diabetic-Sprague Dawley rats have impaired peri-implant bone formation, matrix composition, and implant fixation strength**

Kyle D. Anderson^1^, Christian Beckmann^2^, Saskia Heermant^2^, Frank C. Ko^1,2^, Bryan Dulion^1^, Imad Tarhoni^1^, Jeffrey A. Borgia^1^, Amarjit S. Virdi^1,2^, Markus A. Wimmer^2^, D. Rick Sumner^1,2^, Ryan D. Ross^1,2,3^

1. Department of Anatomy & Cell Biology, Rush University Medical Center, Chicago, IL

2. Department of Orthopedic Surgery, Rush University Medical Center, Chicago, IL

3. Department of Microbial Pathogens and Immunity, Rush University Medical Center, Chicago, IL

**Supplemental Materials**

**
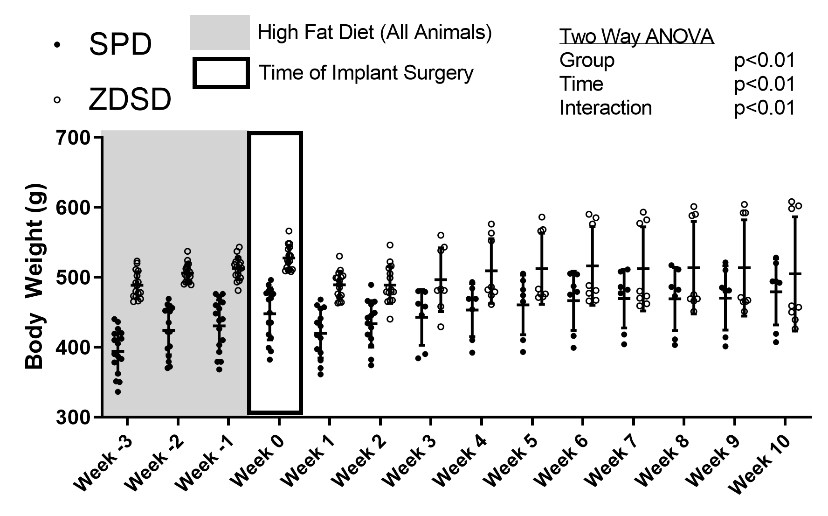
**

**Supplemental Figure 1:** Body weight of Sprague Dawley (SPD) and Zucker Diabetic-Sprague Dawley (ZDSD) groups. Data are reported as the means and standard deviations with each data point representing an individual animal. Week -3 through week 10 represent the experimental timeline with Week 0 representing the time of surgery. The shaded area represents the period at which both groups received high fat diet. Results from a repeated two-way analysis of variance are reported in the legend. Post-hoc differences are not presented within the figure, but ZDSD rats had higher body weight between week -3 and week 4. There were not differences between week 5 and week 10.

**
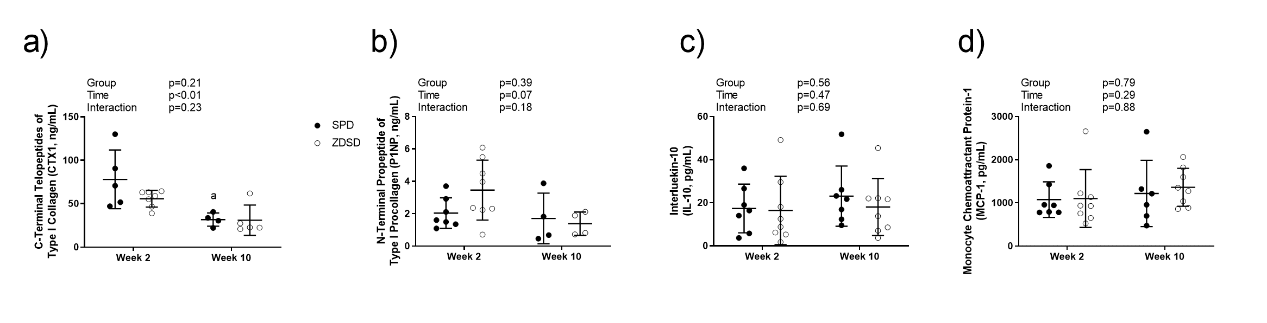
**

**Supplemental Figure 2:** Circulating bone turnover markers and inflammatory cytokines collected at the time of euthanasia – 2- and 10-weeks post implant placement. (a) CTX-1, (b) P1NP, (c) IL-10, and (d) MCP-1 from Sprague Dawley (SPD) and Zucker Diabetic-Sprague Dawley (ZDSD) groups. Data are reported as the means and standard deviations with each data point representing an individual animal. Results from a two-way analysis of variance are reported in the legend. No post-hoc differences were noted.

**Supplemental Table 1:** Variables Significantly Impacted by Diabetic Status

|  | Week 2 | | | Week 10 | | | Two-way ANOVA | | |
| --- | --- | --- | --- | --- | --- | --- | --- | --- | --- |
| Group | SPD | Variable Hyperglycemica | Sustained Hyperglycemia | SPD | Variable Hyperglycemica | Sustained Hyperglycemia | Group | Time | Interaction |
| Fixation Strength | 0.69  (0.37) | **0.05**  **(0.02)*** | **0.10**  **(0.08)*** | 0.59  (0.37) | 1.25  (0.53)b | 0.53  (0.47) | 0.08 | **<0.01** | **0.04** |
| Tb.MAR | 2.13  (0.49) | **3.19**  **(0.75)*** | **2.80**  **(0.39)*** | 1.96  (0.29) | **1.70**  **(0.07)‡** | **1.37**  **(0.20)*^c^** | 0.13 | **<0.01** | **0.01** |
| Tb.BFR/BS | 0.38  (0.16) | 0.56  (0.03) | 0.55  (0.18) | 0.29  (0.15) | 0.23  (0.06)b | **0.12**  **(0.04)*** | 0.68 | **<0.01** | **0.02** |
| Ec.MAR | 3.96  (0.30) | **2.80**  **(0.27)*** | **2.47**  **(0.50)*** | **2.89**  **(0.61)^a^** | 2.45  (0.05) | **0.90**  **(0.56)***^‡^**^c^** | **<0.01** | **<0.01** | 0.06 |
| Ec.MS/BS | 66.83  (10.57) | **45.56**  **(4.19)*** | **43.14**  **(8.96)*** | 58.75  (14.42) | 48.80  (5.93) | **17.82**  **(11.75)*^c^** | **<0.01** | **0.03** | **0.05** |
| Ec. BFR/BS | 2.64  (0.39) | **1.28**  **(0.16)*** | **1.09**  **(0.45)*** | **1.75**  **(0.73)^a^** | **1.20**  **(0.16)** | **0.21**  **(0.19)***^‡^**^c^** | **<0.01** | **0.02** | 0.19 |
| Bone Area | 5.90  (0.49) | 6.29  (0.10) | 6.26  (0.12) | 5.97  (0.78) | 7.24  (0.23)b | 5.73  (0.54) | **0.01** | 0.43 | **0.04** |
| Total Area | 6.21  (0.65) | 6.71  (0.20) | 6.62  (0.13) | 6.10  (0.82) | 7.33  (0.26) | **5.90**  **(0.41)^c^** | **0.01** | 0.75 | 0.08 |
| BV/TV | 0.19  (0.06) | 0.15  (0.04) | 0.14  (0.03) | 0.17  (0.04) | 0.15  (0.02) | **0.10**  **(0.03)***^‡^ | **0.01** | 0.30 | 0.54 |
| Tb.N | 3.53  (0.70) | **2.45**  **(0.61)*** | **2.47**  **(0.17)*** | **2.30**  **(0.77)^a^** | 1.95  (0.37) | **1.86**  **(0.37)^c^** | **<0.01** | **<0.01** | 0.32 |
| Tb.Th | 0.08  (0.02) | 0.09  (0.01) | 0.09  (0.01) | 0.09  (0.01) | 0.09  (0.01) | 0.08  (0.01) | 0.31 | 0.24 | **0.03** |
| Tb.Sp | 0.29  (0.06) | **0.43**  **(0.10)*** | **0.41**  **(0.03)*** | **0.47**  **(0.14)^a^** | 0.53  (0.10) | **0.56**  **(0.11)^c^** | **0.03** | **0.01** | 0.69 |
| Connectivity Density | 85.65 (30.27) | 48.67  (17.58) | **43.26**  **(1.95)*** | **42.45**  **(15.07)^a^** | 25.38  (10.63) | **26.26**  **(12.48)^c^** | **<0.01** | **<0.01** | 0.23 |
| Oss Carb. Sub. (_ν1_PO_4_) | 0.29  (0.07) | 0.28  (0.03) | 0.26  (0.04) | 0.25  (0.04) | **0.34**  **(0.01)*** | 0.36  (0.10) | 0.21 | 0.13 | **0.05** |
| Oss Crystallinity | 18.56  (0.66) | 19.44  (1.46) | 19.15  (1.30) | 17.91  (0.71) | 19.01  (0.95) | **19.43**  **(1.30)*** | **0.02** | 0.72 | 0.35 |
| * indicates significant difference from SPD at the same time  ‡ indicates significant difference from variable hyperglycemia at the same time  Significant post-hoc differences between SPD rats between weeks 2 and 10 are indicated with a bold “a” and “c” indicates differences between weeks 2 and 10 for the sustained hyperglycemia group. There were no significant differences between weeks 2 and 10 for the variable hyperglycemia group. | | | | | | | | | |

**Supplemental Table 2:** :Post-operative P1NP According to Diabetic Status

| Group | SPD | Variable Hyperglycemica | Sustained Hyperglycemia |
| --- | --- | --- | --- |
| P1NP | 2.23  (0.65) | 2.17  (0.66) | **1.19 (0.34)***‡ |
| * indicates significant difference from SPD  ‡ indicates significant difference from variable hyperglycemia | | | |
